# Translation, Cultural Adaptation, and Validation of the Revised Patients' Attitudes Towards Deprescribing Questionnaire for Older Adults Into Greek

**DOI:** 10.7759/cureus.92451

**Published:** 2025-09-16

**Authors:** Alexandros Paraskevopoulos, Björn Wettermark, Izolde Bouloukaki, Ioanna Tsiligianni

**Affiliations:** 1 Department of Social Medicine, School of Medicine, University of Crete, Heraklion, GRC; 2 Department of Pharmacy, Faculty of Pharmacy, Uppsala University, Uppsala, SWE; 3 Department of Primary Care and Population Health, Medical School, University of Nicosia, Nicosia, CYP

**Keywords:** deprescribing, greek language, older adults, psychometric validation, questionnaire translation and adaptation

## Abstract

Background

Deprescribing is a key strategy to combat polypharmacy and enhance clinical outcomes. However, successful deprescribing interventions are significantly influenced by the patient's attitudes, beliefs, and preferences. The aim of this study was to translate and validate into Greek the revised Patients' Attitudes Towards Deprescribing (rPATD) questionnaire, which assesses the attitudes and beliefs of older adults about deprescribing.

Methods

The rPATD questionnaire for older adults was translated using forward-backward translation into Greek. To evaluate psychometric properties, older adults aged ≥65 years taking at least five medications were recruited from 15 primary care facilities in Heraklion, Crete, Greece. Construct validity was examined through exploratory factor analysis (EFA). Reliability was assessed through internal consistency, measured by Cronbach's alpha and item-total correlations, as well as test-retest reliability, determined by the intra-class correlation coefficient (ICC) and weighted kappa coefficient. Floor and ceiling effects were examined.

Results

A total of 255 participants completed the questionnaire. Consistent with the English rPATD, EFA revealed a four-factor structure addressing beliefs about medication appropriateness, perceived medication burden, the level of involvement in medication management, and concerns about stopping medication. Acceptable internal consistency was indicated by Cronbach's alpha values, which ranged from 0.691 to 0.840. Test-retest reliability was satisfactory for all factors, with ICC values ranging from 0.702 to 0.962. A substantial ceiling effect was observed in the involvement factor, while no factor exhibited a significant floor effect.

Conclusions

The Greek adaptation of the rPATD demonstrated psychometric soundness and provides a valid and reliable tool for evaluating attitudes towards deprescribing among older adults.

## Introduction

The global population of older adults is steadily growing, and multimorbidity is common in this group, often requiring complex medication regimens [1]. Polypharmacy, commonly defined as the concurrent use of five or more medications [1], has been shown to increase with age. Data from the Survey of Health, Ageing and Retirement in Europe (SHARE, Wave 6), which included over 68,000 participants from 17 European countries and Israel, revealed that the prevalence of polypharmacy rose from 26.7% among individuals aged 65-74 to 43.1% in those aged 85 and older [2]. Polypharmacy is also associated with many negative clinical outcomes, including elevated risk of adverse drug events, geriatric syndromes, non-adherence, and increased healthcare utilization and costs [1,3]. Interventions prioritizing optimized drug regimens for older adults are therefore strongly recommended [3].

To mitigate the potential adverse effects of polypharmacy, deprescribing has been proposed as a solution. Deprescribing is defined as “the process of withdrawal of an inappropriate medication, supervised by a health care professional with the goal of managing polypharmacy and improving outcomes” [4]. The outcomes of deprescribing have been extensively investigated, with numerous publications highlighting its benefits [5]. Primary care settings, typically led by general practitioners, are well-positioned to manage polypharmacy through continuous, patient-centered care [3]. The involvement of the patient in the process of deprescribing plays a crucial role in optimizing the effectiveness of medication interventions [6]. Patients' ambivalence or reluctance to changes in their medication regimen has been identified as a significant barrier, as perceived by healthcare professionals. Conversely, patients' openness to deprescribing is regarded as a pivotal enabling factor [7].

Examining patients' beliefs about their medications and their attitudes towards deprescribing could provide valuable insights into their willingness to engage in discussions about medication optimization, prior to any recommendations for reducing or discontinuing medications [6]. In 2012, Reeve et al. developed the Patients' Attitudes Towards Deprescribing (PATD) questionnaire, a tool that captures the views of patients regarding their medications and unmasking attitudes that could hinder deprescribing efforts [8]. Subsequently, a revised version, the revised Patients' Attitudes Towards Deprescribing (rPATD), including separate versions for older adults and caregivers, was developed and validated in an Australian population [9].

A systematic review of studies employing the rPATD questionnaire revealed that patients in low- and middle-income countries appear less willing to discontinue their medications than patients in high-income countries. Thus, the attitudes of older adults towards deprescribing are influenced by contextual factors such as the healthcare system, country, or income [10]. To accurately capture these attitudes, it is essential that assessment tools be available in the respondents' native language and culturally validated. To date, the rPATD questionnaire has not been translated or validated into Greek.

In Greece, the population is aging rapidly, with 23% aged 65 and older [11], and polypharmacy is prevalent among older adults [12]. The healthcare system is specialist-focused, with general practitioners comprising only 6% of all physicians, the EU's lowest proportion. Concurrently, out-of-pocket costs for medications are among the highest in Europe [11]. These contextual factors highlight both the importance and the challenges in deprescribing for older adults in Greece. Additionally, a Greek study indicated that general practitioners may be reluctant to deprescribe for older adults due to the perception that these individuals harbor negative attitudes towards discontinuing medications [13]. Research on patient perspectives towards medication use in Greece remains limited, especially in relation to deprescribing. This underscores the need for validated tools to assess these attitudes as a foundation for managing polypharmacy effectively. Therefore, the aim of this study was to translate, cross-culturally adapt, and validate a Greek version of the rPATD questionnaire for older adults in primary care settings.

## Materials and methods

Questionnaire

The English version of the rPATD questionnaire for older adults was developed by Reeve et al. to explore their attitudes and beliefs regarding medication use and deprescribing [9]. It comprises 22 items, including 20 statements corresponding to four factors and two standalone global statements (“Overall, I am satisfied with my current medicines” and “If my doctor said it was possible, I would be willing to stop one or more of my current medicines”). The four factors assessed are (1) belief in appropriateness of medications, (2) perceived burden of their medications, (3) concerns about stopping, and (4) level of involvement in medication management, with each factor represented by five questions. To answer each question, participants have to use a five-point Likert scale (1 = strongly disagree, 2 = disagree, 3 = unsure, 4 = agree, and 5 = strongly agree). Afterwards, the average of the summed score is calculated for each of the four factors and provides a final score from 1 to 5. Global questions do not contribute to the score calculation. Higher scores indicate a greater perceived burden, increased concerns about stopping, a stronger belief in the appropriateness of their medications (due to reverse scoring of the corresponding questions), and a higher level of involvement [9].

Translation and cross-cultural adaptation

Permission to translate, adapt, and validate the rPATD questionnaire was granted by the primary author of the original instrument [9]. The translation and cultural adaptation process followed the recommendations of the Professional Society for Health Economics and Outcomes Research (ISPOR) Task Force for Translation and Cultural Adaptation [14] as a guiding framework, with certain steps adapted to local feasibility. The process was completed in five stages. In the first stage, the English version of the rPATD questionnaire for older adults was translated into Greek through a forward translation procedure. Two bilingual translators (AP and AK), both native Greek speakers fluent in English and with a medical background, independently performed the translations. A reconciliation phase followed, in which the two translators and a bilingual professor (IT) collaborated to address discrepancies in the initial translations, resulting in a unified final translation. The third stage involved a back-translation process. The final forward translation was back-translated into English by a third bilingual translator (AC) who was a native Greek speaker, fluent in English, and also had a medical background. During the fourth stage, a team was formed to harmonize the various versions of the translated questionnaire. This team consisted of the two forward translators, the back-translator, and two bilingual professors (IT and IB). Together, they carefully reviewed all versions and resolved any inconsistencies to achieve the final Greek version. Finally, to assess face and content validity, the finalized Greek version was tested with a separate sample of adult patients aged 65 years or older who were taking at least five medications. The participants were given the questionnaire for self-administration, and face and content validity were assessed through cognitive debriefing [14]. A researcher (AP) was present during questionnaire administration and conducted the cognitive interviews. Probing questions were asked after each question to ensure that participants understood the items, and they were encouraged to verbalize any questions that came up while completing the questionnaire. The comprehension and reactions of the pre-test participants were evaluated. As a result, minor improvements were made to enhance clarity in the Greek language, but no major revisions were necessary.

Recruitment and data collection

A multicenter cross-sectional study was conducted across 15 primary care facilities (six urban and nine rural) in Heraklion, Crete, Greece, between November 15, 2024, and December 20, 2024. The nine rural facilities included five primary care centers and four regional clinics affiliated with these centers. Primary care centers served large numbers of patients, typically employing multidisciplinary teams of general practitioners, nurses, and other health professionals. In contrast, regional clinics served fewer patients and were doctor-led, supported by a limited number of nurses. Recruitment for the study was conducted by two researchers (AP and AT) who visited primary care facilities and screened older adults who were waiting for their scheduled visit. The selection criteria for eligibility included being 65 years of age or older, residing in the community, taking a minimum of five chronic medications (prescribed for three or more months), and being able to read, understand, and sign a consent form. Older adults who met the inclusion criteria were briefed about the study objectives and were invited to participate. Those who accepted were given a consent form to sign. The researchers administered the questionnaire to the participants by reciting the statements of the questionnaire and then asking them to indicate their level of agreement using the five-point Likert scale. Participants were also requested to provide demographic information, such as age, gender, and number of chronic medications. Participants were also evaluated for frailty using the Clinical Frailty Scale (CFS), with data derived from their clinical records in consultation with their doctors. The CFS is a nine-point scale that assesses an individual's overall fitness or frailty. Scores one to three indicate no frailty, a score of four reflects very mild frailty, while scores of five to nine correspond to increasing frailty [15].

Sample size calculation

There is no universal consensus on the appropriate method for calculating sample size in validation studies of questionnaires. However, the sample size should be 100 or larger, and an acceptable size would have a 10:1 ratio of participants to the number of variables to be analyzed [16]. Therefore, a minimum sample size of 220 older adults was deemed appropriate since the older adults' version of the rPATD questionnaire contains 22 statements.

Psychometric properties

The psychometric evaluation of the Greek version of the rPATD questionnaire for older adults included the assessment of content and face validity, construct validity, and reliability to ensure the translated version maintains conceptual and measurement equivalence with the original instrument. To assess the acceptability of the questionnaire, the proportion of missing responses was analyzed. Missing data levels below 10% are generally deemed acceptable [16]. For psychometric analyses, cases with missing responses were excluded listwise. All statistical analyses were conducted using IBM SPSS Statistics version 26.0 (IBM Corporation, Armonk, NY, USA) for Windows. A type I error rate of <0.05 was considered significant.

Content and Face Validity

Content and face validity were evaluated in the translation and cross-cultural adaptation process, during a pre-test phase of the final Greek version of the questionnaire, as aforementioned, with older adults to evaluate the comprehension of the questionnaire items and confirm conceptual equivalence and linguistic clarity.

Construct Validity

An exploratory factor analysis (EFA) was carried out to investigate the factor structure of the instrument and to determine whether additional dimensions specific to the Greek population emerged. This analysis assessed structural validity, which refers to the extent to which instrument scores reflect the dimensional structure of the construct being assessed [17], thus providing evidence for construct validity. To assess the dataset's suitability for EFA, we employed the Kaiser-Meyer-Olkin (KMO) measure of sampling adequacy and Bartlett's test of sphericity. KMO values ≥0.70 are desired, and values <0.5 are considered unacceptable [18]. Bartlett's test assesses whether the correlation matrix differs from an identity matrix. A significant p-value suggests that the correlation matrix is non-random, indicating the suitability of factor analysis for the dataset [18]. The Kaiser-Guttman eigenvalue-greater-than-one rule and scree plots guided our decision on the number of factors to retain [19]. Promax rotation was employed to enhance the interpretability of the factor structure while allowing for potential correlations among factors [19]. While factor loadings >0.30 are considered acceptable, we applied a more stringent threshold of ±0.35, following Hair et al., who suggested that this level is statistically significant with samples of around 250 participants [16].

Reliability

The reliability of the Greek version of the rPATD was assessed through internal consistency and test-retest reliability. Internal consistency was evaluated using Cronbach's alpha (α), with a threshold of ≥0.70 considered satisfactory. Values between 0.60 and <0.70 are generally viewed as questionable, whereas values below 0.60 indicate weak internal consistency. Additionally, item-total correlations were calculated, with values >0.30 considered acceptable [20]. Test-retest reliability was evaluated by administering the questionnaire to a subset of participants twice, with a two-week interval, and calculating the intra-class correlation coefficient (ICC) and the weighted kappa coefficient. Weighted kappa values were interpreted as follows: 0.00-0.20 (slight agreement), 0.21-0.40 (fair), 0.41-0.60 (moderate), 0.61-0.80 (substantial), and >0.80 (almost perfect). In the weighted kappa calculation, quadratic weights were used to penalize disagreements between adjacent categories less than those between more distant ones [21]. An ICC value of ≥0.70 was considered indicative of acceptable reliability [22].

Floor and ceiling effects

The floor and ceiling effects were assessed for each individual factor by calculating the percentage of participants who achieved the lowest or highest possible scores. A threshold of <15% was considered acceptable [22].

Ethics

Ethical approval for the study was obtained from the Research Ethics Committee of the University of Crete (approval number 93/28.06.2023). In addition, permission to conduct the research in the primary care facilities was granted by the Seventh Health Region of Greece (approval number 16888/17-4-24). All participants voluntarily engaged in the study after providing written informed consent. Additionally, all data were anonymized to ensure confidentiality and privacy.

## Results

Translation and cross-cultural adaptation

The Greek rPATD questionnaire for older adults was translated and cross-culturally adapted following the procedures outlined in the Materials and Methods section. During the pre-test phase, the questionnaire was tested on a sample of 16 adult patients aged 65 years or older, all of whom were taking at least five medications. Participants for the pre-test phase were recruited using convenience sampling from urban primary care facilities. This phase aimed to assess the content and face validity of the instrument. Overall, the participants demonstrated a clear understanding of the questionnaire items. However, some required additional clarification regarding the term “inconvenient” (Q9: “Taking my medicines every day is very inconvenient”). To address this, both the terms “inconvenient” and “difficult” were used during the administration of this question. Other changes that occurred during this pre-test phase included only minor adjustments to the wording of some questionnaire items to enhance clarity and comprehension. The final version of the Greek rPATD questionnaire for older adults is available from the corresponding author upon reasonable request and with permission from the original instrument developers [9].

Participants' characteristics

A total of 255 older adults completed the questionnaire. There was an equal distribution between genders, and the median age of the participants was 76 years (IQR: 71-80). The median number of prescribed medications among participants was nine (IQR: 7-11), while a significant portion (40.4%) were prescribed at least 10 medicines. Missing items were identified in only three questions (Q10, Q14, Q15), with a rate ranging from 0.39% to 0.78%, which was considered low and acceptable [16]. The characteristics of the participants are presented in Table 1.

**Table 1:** Characteristics of study participants. IQR: interquartile range.

Characteristic		Value
Gender		
Female, n (%)	130(51%)	
Male, n (%)	125(49%)	
Age (years), Median (IQR)	76(71-80)	
Residence		
Urban, n (%)	134(52.5%)	
Rural, n (%)	121(47.5%)	
Number of prescribed medications		
Median (IQR)	9(7-11)	
5-9, n (%)	152(59.6%)	
10-14, n (%)	88(34.5%)	
≥15, n (%)	15(5.9%)	
Clinical Frailty Score (CFS)		
No frailty (CFS 1-3), n (%)	135(52.9%)	
Vulnerable/very mild frailty (CFS 4), n (%)	65(25.5%)	
Frailty (CFS≥5), n (%)	55(21.6%)	

Psychometric properties

Construct Validity

To assess construct validity using EFA, we first confirmed the adequacy of our sample through KMO (0.760) and Bartlett's test (p<0.001), which indicated factorability. The EFA was conducted on 20 items of the questionnaire, excluding the two global questions, similar to the original rPATD [9]. After reviewing the eigenvalues and scree plot, we retained four factors that accounted for 48.287% of variance (18.338%, 17.085%, 7.513%, and 5.351%, respectively). All four factors met the Kaiser-Guttman criterion with eigenvalues >1. Although a fifth factor also had an eigenvalue slightly above one, it was located in the scree plot's flattening part. Therefore, we opted for a four-factor solution, aligning with the English rPATD questionnaire [9]. We conducted an EFA with a principal axis factoring extraction method, including a four-factor pre-selection and utilizing promax rotation. All factor loadings exceeded the 0.35 threshold, with no evidence of cross-loading. The pattern of item loadings mirrored the four factors of the original rPATD-involvement, burden, appropriateness, and concerns about stopping (Table 2).

**Table 2:** Psychometric properties of the Greek rPATD questionnaire for older adults. CI: confidence interval; rPATD: revised Patients' Attitudes Towards Deprescribing; Q: question; NA: not applicable. ^a^Internal consistency was assessed with Cronbach's α (95% CI) at the factor level and item–total correlations for questions. ^b^Test-retest reliability was assessed using intra-class correlation coefficients (95% CI) based on a two-way random-effects model with absolute agreement for factor scores, and quadratic weighted kappa coefficients for questions.

Item	Factor loading		Internal consistency^a^	Test-retest reliability^b^
Involvement factor		0.801 (0.760-0.837)		0.917 (0.787-0.967)
Q2 (involved in decisions)	0.419	0.538		0.808
Q3 (good understanding)	0.763	0.564		0.937
Q4 (know as much as possible)	0.751	0.741		0.875
Q5 (always ask if I don't understand)	0.740	0.619		0.929
Q6 (know current medicines)	0.740	0.567		0.750
Burden factor		0.840 (0.807-0.869)		0.885 (0.737-0.952)
Q8 (large number of medicines)	0.862	0.744		0.840
Q9 (inconvenient)	0.682	0.659		0.873
Q10 (money)	0.483	0.394		0.763
Q11 (too many medicines)	0.869	0.762		0.942
Q12 (burden)	0.700	0.677		0.819
Appropriateness factor		0.729 (0.672-0.778)		0.962 (0.879-0.986)
Q13 (would like to try stopping)	0.468	0.528		0.765
Q14 (reduce the dose)	0.391	0.495		0.880
Q15 (medicines that I no longer need)	0.853	0.636		0.907
Q16 (side effects)	0.431	0.320		0.882
Q17 (not working)	0.793	0.532		0.886
Concerns about stopping factor		0.691 (0.627-0.747)		0.702 (0.385-0.871)
Q18 (previous bad experience)	0.407	0.314		0.766
Q19 (reluctant to stop a long-term medicine)	0.721	0.548		0.275
Q20 (missing out on future benefits)	0.844	0.635		0.959
Q21 (stressed)	0.465	0.326		0.536
Q22 (giving up)	0.483	0.487		0.750
Global questions				
Q1 (satisfaction)	NA	NA		0.824
Q7 (willing to stop)	NA	NA		1.000

Internal Consistency

Corrected item-total correlations for all items were above the recommended 0.30 threshold [20]. Internal consistency was satisfactory for the burden (α = 0.840), appropriateness (α = 0.729), and involvement (α = 0.801) factors (Table 2). However, the concerns about stopping factor showed a lower coefficient (α = 0.691), indicating questionable internal consistency [20].

Test-Retest Reliability

To evaluate the test-retest reliability, a subset of 20 participants was chosen from the total study sample, based on their availability and ability to be contacted by phone. Out of the 20 participants, 15 were female and five were male, with a median age of 73.5 years (IQR: 69-77.5). Each participant completed the questionnaire for a second time between two and three weeks after their initial response. Phone interviews were conducted to obtain the second round of responses. All factors demonstrated satisfactory reliability, with ICC values ranging from 0.702 to 0.962 (Table 2). The results for the weighted kappa coefficient showed variability, ranging from 0.275 to 1.000.

Floor and ceiling effects

Floor and ceiling effects were evaluated for each factor (Table 3). There was no evidence of a significant floor effect. A substantial ceiling effect was observed in the involvement factor, with 16.86% of participants achieving the highest possible score.

**Table 3:** Floor and ceiling effects of the Greek rPATD questionnaire for older adults. rPATD: revised Patients' Attitudes Towards Deprescribing.

Factor	Skewness	Floor effect (%)	Ceiling effect (%)
Involvement factor	-0.322	0.0	16.86
Burden factor	-0.057	1.19	5.14
Appropriateness factor	-0.088	0.0	4.74
Concerns about stopping factor	0.183	10.59	0.0

## Discussion

The rPATD questionnaire was adapted into Greek for use with older adults through established procedures [14]. The final version showed satisfactory psychometric properties, supported by evidence from content and face validity assessments, as well as construct validity and reliability evaluations.

The exploratory factor analysis (EFA) initially indicated a total of five factors, according to the Kaiser-Guttman criterion. While the Kaiser-Guttman criterion is widely used due to its simplicity, it has limitations. Specifically, it may overestimate the number of factors to retain [19]. To overcome this limitation, the scree test is recommended. This involves analyzing the eigenvalue plot to identify the inflection point, where the curve flattens, and retaining the factors corresponding to the data points above this “break”. The selection can be refined by running multiple factor analyses and choosing the solution with the “clearest” factor structure [19]. This led to the identification of a four-factor structure, which aligns with both the original rPATD [9] and other validated versions, including the French [23], Romanian [24], and Portuguese [25] translations of the rPATD. All factor loadings were satisfactory, exceeding the 0.35 threshold.

Regarding the internal consistency, the Cronbach's alpha values ranged from 0.691 to 0.840. Similar to the original version [9], where burden and appropriateness factors both scored 0.809, our version showed the highest Cronbach's alpha (0.840) for the burden factor. However, the concerns about stopping factor displayed an unsatisfactory internal consistency with a Cronbach's alpha value of 0.691. Our results align with the original version [9] as well as with the French [23], Romanian [24], and Portuguese [25] versions, all of which exhibited unsatisfactory internal consistency for the concerns about stopping factor. However, consistent with the aforementioned versions, the concerns about stopping factor internal consistency was considered adequate, as values of Cronbach's alpha ranging from 0.6 to 0.7 indicate acceptable reliability [26]. Reeve et al. suggested that the lower internal consistency of the concerns about stopping factor may be due to the absence of a single underlying belief that affects how all the questions in this factor are answered [9].

The appropriateness (ICC = 0.962), involvement (ICC = 0.917), and burden (ICC = 0.885) factors exhibited high ICC values, indicating satisfactory test-retest reliability. Although the concerns about stopping factor had the lowest ICC (0.702), it still exceeded the predefined threshold of 0.70, reflecting acceptable reliability [22]. Our results align with previous research on older adults using the French rPATD questionnaire [23], where “concerns about stopping” showed the lowest ICC. In our study, the test-retest results showed that most changes in participant responses were minor shifts between similar agreement levels (e.g., “Agree” and “Absolutely agree”). During the evaluation of the weighted kappa coefficients, Q19 (reluctant to stop a long-term medicine) had the lowest score of 0.275. Upon closer examination, it was observed that most participants' responses shifted in the same direction, such as from “absolutely agree” to “agree”, except for one participant who had a significant change from “absolutely agree” to “absolutely disagree”. This extreme shift impacted the low score, as it was heavily penalized in the quadratic weighted kappa calculation. In contrast, the linear weighted kappa calculation resulted in an increased coefficient of 0.459.

During the assessment of ceiling and floor effects, it was found that only the involvement factor displayed a notable ceiling effect of 16.86%. The French, Romanian, and Portuguese versions [23-25] also reported ceiling effects exceeding 15% for the involvement factor. The French and Romanian validation studies [23,24] further suggested that the use of a 15% threshold may be too strict for the rPATD questionnaire, given its use of five-point Likert items and factor scores with a possible range of 1-5. Moreover, response styles [27], such as social desirability bias [21] and acquiescent responding, which may be more common among older individuals [28], could be a contributing factor to the ceiling effect seen in our study.

Strengths and limitations

Our study benefits from a structured approach to translation, cultural adaptation, and psychometric evaluation, informed by established guidelines and recommended practices [14,20,22]. Another strength of this study is the inclusion of only older adults with polypharmacy, who are in greater need of deprescribing as a high-risk group. Additionally, a substantial portion (47.1%) of the study population was identified as pre-frail or frail, a group particularly susceptible to the negative health effects of polypharmacy [1].

A limitation of this study is that during data collection, the questionnaire was administered by a researcher, whose presence may have induced the social desirability bias [21]. Another limitation is that our study specifically recruited older adults in Heraklion, Crete, Greece. Therefore, the generalizability of our results to different regions, settings, or populations may be limited. While the translation and adaptation process was guided by ISPOR recommendations [14], some adaptations were made for feasibility. Most notably, the back translator was native in the target rather than the source language, which was addressed through blinding of the back translator and item-by-item review of the back-translation against the original English version. Furthermore, the criterion validity of the adapted rPATD questionnaire was not assessed because there isn't a sufficient gold-standard counterpart in Greek. Although the Beliefs about Medicines Questionnaire (BMQ) has been utilized in previous rPATD validation studies to assess concurrent validity, and a Greek validated version exists [29], it was not incorporated in our study. Moreover, our study did not include a translation or adaptation of the caregivers' version of the rPATD. Future research should adapt and validate the caregiver version of the rPATD for Greek-speaking caregivers.

## Conclusions

The Greek version of the rPATD for older adults exhibited a four-factor structure, consistent with the original rPATD, and demonstrated satisfactory psychometric properties. This tool could be used to facilitate deprescribing discussions and inform healthcare providers about patients' potential resistance or receptiveness. The questionnaire could also be used by researchers to guide the design of future interventions tailored to the specific needs of older adults. The Greek version of the rPATD questionnaire for older adults is applicable in both clinical settings and research, serving as a valuable tool for addressing polypharmacy and supporting deprescribing efforts.
